# The Influence of Social Determinants of Health on the Survival of Heart Transplants in the Pediatric Age: An Analysis of a Mexican Cohort and Its Comparison with Latin America and the Caribbean

**DOI:** 10.3390/jcm14051506

**Published:** 2025-02-24

**Authors:** Horacio Márquez-González, Alejandro Bolio-Cerdán, Sergio Ruiz-González, Julio Erdmenger-Orellana, Carlos Alcántara-Noguez, Ma Pueblito Patricia Romero-Cárdenas, Diana Avila-Montiel, Solange Gabriela Koretzky

**Affiliations:** 1 Department of Clinical Research, Federico Gómez Children’s Hospital, Mexico City 06720, Mexico; hmarquez@himfg.edu.mx (H.M.-G.); diana.avmo@gmail.com (D.A.-M.); 2 Department of Cardiothoracic Surgery, Federico Gómez Children’s Hospital, Mexico City 06720, Mexico; dr.alexbolio@gmail.com (A.B.-C.); dr.charlyalcantara@gmail.com (C.A.-N.); paty_ro_ca@hotmail.com (M.P.P.R.-C.); 3 Sub Directorate of Surgical Assistance, Federico Gómez Children’s Hospital, Mexico City 06720, Mexico; subdireccionquirurgica@himfg.edu.mx; 4 Department of Pediatric Cardiology, Federico Gómez Children’s Hospital, Mexico City 06720, Mexico; erdmenger@gmail.com

**Keywords:** heart transplantation, congenital heart disease, pediatric cardiac surgery, pediatric cardiology, children

## Abstract

**Background/Objectives:** A heart transplantation (HT) is the definitive treatment for heart failure. There is a difference in the success between national HT programs in developed countries and those in Central America, South America, and the Caribbean (LAC), and social determinants of health (SDHs) can directly influence this. The objectives of this study were to describe the survival since the beginning of the HT program of a national pediatric institute in Mexico City and to compare it with the results of a systematic review of LAC. **Methods:** A cohort study of a pediatric hospital (which performed 42% of the pediatric HTs in Mexico) was performed since the beginning of the HTs program in 2001. Clinical variables related to the transplants were identified, and the SDHs were divided into three categories: personal, family, and community. A systematic literature review was performed using keywords and a search in the medical indexes of LAC countries. The statistical analysis included descriptive statistics and a bivariate survival analysis. A risk calculation was estimated using the hazard ratio (HR) of the SDHs. **Results:** A total of 38 HTs were performed, the median age was 7 (4–16) years, and 22 (58%) were men. The leading cause was cardiomyopathy in 20 (53%) cases. The first-year survival rate was 76.3 per 100 HTs. The SDHs that increased the risk of death were suboptimal immunosuppression, the persistence of malnutrition, parental education, the distance from the center, the socioeconomic level, and the absence of transitional care. **Conclusions:** This cohort of pediatric patients with HTs resulted in the identification of risk variables of personal and community SDHs for mortality in the first and fifth years. Chronic rejection occurred in 50%, and the absence of transitional care to adulthood was the variable with the highest risk. The systematic review identified Mexico as the country with the second-highest frequency of HTs, and our cohort represented 42% of the total number of transplants in the country. Numerically, LAC has a lower frequency of transplants and survival in the first year compared to other developed countries, possibly due to a gap associated with organizational justice.

## 1. Introduction

A heart transplantation (HT) is the final stage of treating heart failure (HF). Since Dr. Christian Barnard successfully performed it in 1967, there has been exponential and specialized growth in the surgical, intensive care, and pharmacological treatment areas to support the function of the transplanted organ. These advancements have also extended to the pediatric stage, which has apparent differences compared to other life periods, such as the etiology, access to the organ, and the effects of growth and development on the function of the transplanted heart [1].

In addition to trained health personnel, an organized national program is needed to ensure the fair distribution of available organs, the proper selection of the best candidates, and multidisciplinary support throughout all phases [2,3].

In ideal conditions, the results of heart transplantation during the pediatric stage are like those seen in adults. The ISHLT (International Society of Heart and Lung Transplantation) registry, composed of data from 210 centers, reported that, from 1992 to 2018, 13,449 pediatric patients received an HT. According to the annual report, in 2022, 80% of transplants were performed in the United States of America (USA); the total population included 2109 children, with a first-year survival rate of 73%. Less than 10% were in emergency conditions due to ventricular support or ECMO (extracorporeal membrane oxygenation), 0.3% were retransplants, and acute rejection occurred in 3% [4].

Transplant programs indirectly reflect the situation in their countries, given their close relationship with the healthcare system, the necessary economic investment, and the social and cultural conditions involved across all areas. For this reason, transplant programs are not a priority in countries with emerging economies compared to other more pressing issues. The American continent, excluding the USA and Canada, has decades of lag in the total numbers and success rates of pediatric HTs [5].

Social determinants of health (SDHs) encompass a range of variables that center the individual and analyze how different social conditions influence their context, either reinforcing or exposing them to vulnerability [6]. Thus, the outcomes of a disease, despite being treated under ideal conditions, can vary drastically because of an adverse context [7,8].

Among Central American, South American, and Caribbean (LAC) countries, Mexico represents the fourteenth-largest economy in the world. It has a consolidated HT program (CENATRA, which stands for Centro Nacional de Trasplantes), which, since its inception in 2001, has managed to perform transplants in just over fifty children, of which 47% were performed at a pediatric hospital in Mexico City, making its results a helpful comparison for the reality of Mexico [9,10]. This article had two main objectives: (a) to describe the survival of pediatric patients who received an HT at a national institute in Mexico and associate the effect of SDHs with mortality in the first and fifth years, and (b) to perform a systematic review of the literature on the status of HTs in LAC.

## 2. Materials and Methods

To address the aims, a preliminary phase involved a cohort study of patients under 18 years old who underwent heart transplants at a pediatric national health institute in Mexico City, starting from their entry into the national transplant program (2001) until December 2023. This study was conducted according to the guidelines of the Declaration of Helsinki and approved by the Institutional Review Board and Ethics Committee of the Federico Gómez Children’s Hospital of Mexico (protocol code HIM-2022-042, date of approval: 29 November 2022). The data were obtained from medical records and information from institutional databases, which were anonymized during the analysis. This retrospective work is classified as “risk-free research”, so it does not require an informed consent letter, only a process to ensure that the research subjects are not identified during the data analysis and publication.

The selection of subjects included all the patients transplanted since the program’s start, with annual follow-ups until they turned 18. Patients who were transplanted at another hospital or were lost to follow-up for more than a year were excluded. The index date of the cohort was defined as the hospital discharge after the transplant or the achievement of 30 days of survival. The primary outcome variable was survival in the first, fifth, and tenth years. Follow-up was ensured through physical and electronic records and social work follow-up calls.

As a reference, we took the Dahlgren–Whitehead model in the operational framework for monitoring social determinants of health equity [11]. For the first sphere of the general characteristics, we included clinical variables such as the sex, age in months, and underlying condition, categorized in two ways: (1) cardiomyopathies, including the dilated, hypertrophic, asymmetric septal, and constrictive types, and (2) advanced congenital heart diseases (CHDs) with heart failure. The blood type and anthropometric measures (weight in kilograms, height in centimeters, body surface area, and body mass index) were also noted. Information about cold ischemia times, extracorporeal circulation, aortic clamping, the electrical rhythm at discharge, the number of days in intensive care, and the length of the hospital stay was obtained from the official CENATRA registry. The risk factors considered were divided into the following areas.

The second sphere of the Dahlgren–Whitehead model refers to the individual life factor. We included the waiting time between acceptance into the transplant program and the heart transplant, which was in months. The urgency of the transplant was considered if bridge therapy (ECMO or ventricular support) occurred. The time when the transplant period occurred (2001–2005, 2006–2010, 2011–2015, or 2016–2023) was also included. Inadequate adherence to immunosuppressive medication or pharmacological treatment was noted when mentioned in the records by any of the involved treatment services. Rejection events were documented with histopathological studies. The biopsy results defined the rejection variable according to the ISHLT severity scale for acute cellular rejection [12], as per our institutional protocol; heart transplant recipients undergo endomyocardial biopsies at specific time points: during the first week, first month, second month, sixth month, and first year post-transplant. For this study, we reported the findings of the last biopsy performed during the follow-up period. The nutritional status was determined by the body mass index (BMI) and considered altered when the following conditions were met: less than two Z-score deviations on the World Health Organization (WHO) growth curves one year post-transplant, and weight gain of less than one Z-score deviation compared to the pre-transplant status at one year. The presence of anxiety or depression was recorded by mental health departments, along with the return to school or regular activities [13].

For the social and community determinants, we included the lack of transitional care in adulthood, defined as the absence of attention in hospital settings with adult HT programs, as a risk factor. This also includes limited access to social security or health insurance upon reaching adulthood.

For the sphere of living and working conditions, we included family risk determinants, such as the distance between home and the hospital (categorized as <20 km, 21–100 km, 101–300 km, or 300 km); the education level of the primary caregiver (elementary, middle, high school, technical, or college), and the socioeconomic status, classified according to the INEGI (National Institute of Statistics and Geography) based on monthly income (converted to USA dollars as follows: A, over USD 5500; B, <USD 1666; C, <USD 2700; D, between USD 250 and 1500; and E, less than USD 500). Informal employment was identified as trade or inconsistent income from resources [14].

The second aim was addressed through a systematic literature review to understand heart transplant survival in the pediatric population of LAC, based on PRISMA 2020 statements [15]. Articles (in English, Spanish, and Portuguese) and abstracts that met our PIO format criteria were included.

- Population (P): pediatric patients aged 0 to 18 years with end-stage heart failure or congenital defects.
- Intervention (I): heart transplant.
- Outcome (O): death during the postoperative period.

The inclusion criteria included the following: Observational articles (longitudinal, case-control, and prospective and retrospective cohort) were selected that featured pediatric patients who underwent heart transplants in Latin America or the Caribbean and reported the number of heart transplants, the etiology, the survival, and the cause of death, in Figure S1: are the complete search strategies. Care was taken to select articles that met the established standards outlined in the Strengthening the Reporting of Observational Studies in Epidemiology (STROBE) statement [16].

Publications by the same author at the same hospital were included if they did not fall within the same evaluation period. The exclusion criteria were reports involving patients aged 18 and older who underwent heart transplants and articles that did not report on patient survival. If the articles met the selection criteria, but the data were insufficient for the analysis, the corresponding authors were emailed to request clarification. If they provided the information, it was included in the review.

The sources of information used were PUBMED, the TRIP database, the International Clinical Trials Registry Platform (WHO), The Cochrane Library, Wiley, LILACS, SciELO, and Google Scholar. Systematic reviews related to the objectives were also searched (snowball method). Searches were conducted using the reference lists of the full-text articles to identify additional relevant studies; the search period was extended to 1 December 2024. For reviewing the information, four researchers independently reviewed all the references identified through the literature search using a predefined protocol. Articles that did not meet the inclusion criteria were excluded during the title and abstract analysis. The remaining articles were selected for a full-text review. The full text was always obtained whenever there was limited information in the abstract. All the researchers performed a quality assessment on the articles included. Disagreements regarding the selection and quality evaluation of the articles were resolved through group discussions, achieving a total consensus at each review stage.

The four researchers independently extracted data from the selected studies using a standardized electronic form in Excel. They collected the following information: author, year of publication, country, study design, total number of pediatric heart transplant patients, etiology, and early and late deaths.

They assessed the risk of bias in individual studies, the publication bias, and the certainty of the evidence. To reduce bias, each article was reviewed by at least two authors.

### Statistical Analysis

Descriptive statistics were conducted. Qualitative variables were expressed in absolute numbers and percentages, while quantitative variables did not show a parametric distribution, so they were represented by medians and interquartile ranges (25th–75th percentiles).

In inferential statistics, comparison groups were created for the following periods: 2001–2005, 2006–2010, 2011–2015, and 2016–2022. Statistical differences were estimated using the Wilcoxon rank test.

The risk calculation between the variables that assessed SDHs and the mortality at 1 and 5 years post-HT was performed using a hazard ratio (HR) adjusted for the etiology of heart failure, sex, and age. Using a forward conditional approach, a Cox regression analysis was conducted following the specified criteria, including an initial inclusion threshold of a *p*-value < 0.1, clinical relevance, and prior literature evidence. Variance inflation factors (VIFs) were used to assess the collinearity, and highly correlated variables were removed or combined to improve the model stability and interpretability. The final model included adherence to immunosuppressive therapy, optimal heart failure management, persistent malnutrition at one year, depression, return to school, distance to the transplant center, caregiver education, socioeconomic status (INEGI classification), and informal employment. However, due to the sample size, the number of variables, and collinearity issues, it was impossible to complete the Cox regression analysis. Therefore, only unadjusted hazard ratios (HRs) are reported.

For this reason, multiple Cox regression models were calculated to calculate the HR value of each of the variables separately, adjusting for the etiology of heart failure, sex, and age at transplantation for death in the first and fifth years.

The program used was SPSS, version 29, for IBM on Mac.

## 3. Results

Since the HT program started in 2001 at the institution where the study was conducted, 123 patients were enrolled, and 38 transplants were performed. The median age was seven years (4 to 16), and 22 (58%) were male. Cardiomyopathies were the leading cause of heart failure in 20 (53%) cases. Among these, 14 cases (70%) were due to dilated cardiomyopathy secondary to myocarditis, 5 cases (25%) were due to hypertrophic cardiomyopathy, and 1 case (5%) was due to restrictive cardiomyopathy. The one-year mortality rate was 21 out of every 100 transplants, with the leading causes being (*n* = 9) heart failure in five (55.5%) patients and septic shock in one (11.1%). Among these patients, four (44%) experienced acute rejection (Table 1). Of the total cohort, 19 (50%) presented with transplant rejection, of which 10 (52.6%) were grade IR, 7 (36.8%) were grade 2R, and 2 (10.5%) were grade 3R.

Regarding the surgical variables (Table 2), when comparing the survivor group to the non-survivors after the first year, statistically significant differences were found for the cold ischemia time in minutes (169 (165–250) vs. 275 (235–315)) and the extracorporeal bypass time in minutes (148 (125–160) vs. 123 (115–150)).

In the analysis of personal SDHs (Table 3), 24 (63.2%) had an optimal adherence to immunosuppression at discharge and 33 (86.8%) received optimal treatment for heart failure. In 19 (50%), the transplant was rejected. By the end of the first year, 14 (36.8%) continued to experience malnutrition, and 25 (65.8%) returned to school.

In terms of family factors, 16 (42.1%) patients lived less than 50 km away from the hospital; the most common education level of caregivers was higher education in 15 (39.5%) cases; and in 26 (68.4%) of the cases, the socioeconomic level was classified as level D according to the INEGI (for its acronym in Spanish: the National Institute of Statistics, Geography, and Informatics).

Regarding the continuity of healthcare services, 13 (34.2%) received transitional care to adulthood and 10 (26.3%) had social security.

**Figure 1:**
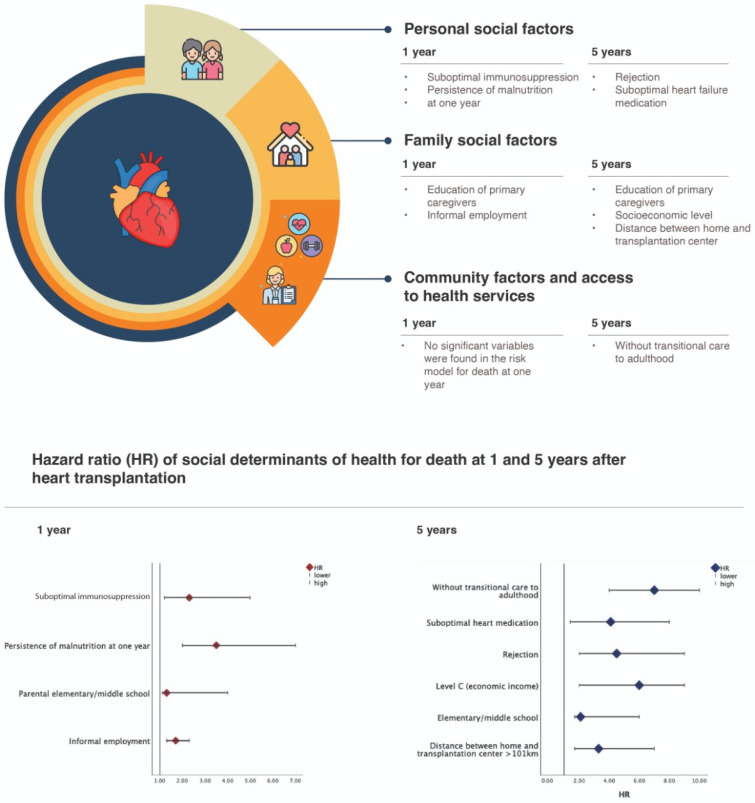
Social determinants of health that have an impact on the survival at 1 and 5 years of pediatric patients receiving an HT.

When adjusting for confounding variables (etiology, sex, age), the factors most associated with death during the first year were suboptimal immunosuppression, malnutrition in the first year, and low parental education; at five years, the factors included rejection, a distance from the center greater than 101 km, suboptimal heart failure medication, the socioeconomic level, and a lack of transitional care to adulthood (Figure 1).

**Figure 2:**
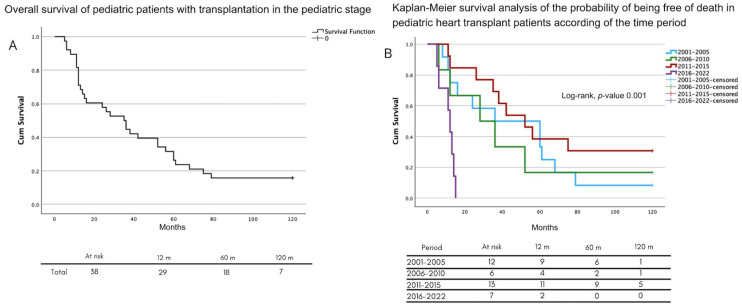
(**A**) Overall survival of pediatric patients who received an HT. (**B**) Analysis of survival of pediatric patients who received an HT by period.

The survival analysis over time showed lower survival rates in the last period (2016–2022) compared to the previous three (Figure 2A), with half of the deaths occurring within 36 months (30–40 months) (Figure 2B). The one-year survival rate was 29 (76.3 per 100 HTs) in the first year, 18 (47.3 per 100 HTs) at five years, and 6 (15.7 per 100 HTs) at ten years.

All the patients in the last period (2016–2022) died within 5 years. During the previous period (2016–2022), most deaths (*n* = 5) occurred during the first year. From 2016 to 2019, three patients received a transplant, of which one patient’s death was attributed to a high surgical complexity (history of Fontan surgery); one was classified as a high priority for urgency without bridging therapy; and one was attributed to a prolonged cold ischemia time secondary to distance from the donor.

From 2019 to 2022, the hospital modified the care priority of the intensive care units to care for the most severe cases of children with SARS-CoV-2 in Mexico City, which led to a decrease in the volume of transplants, and the two patients transplanted at this stage experienced a worsening state of heart failure.

**Figure 3:**
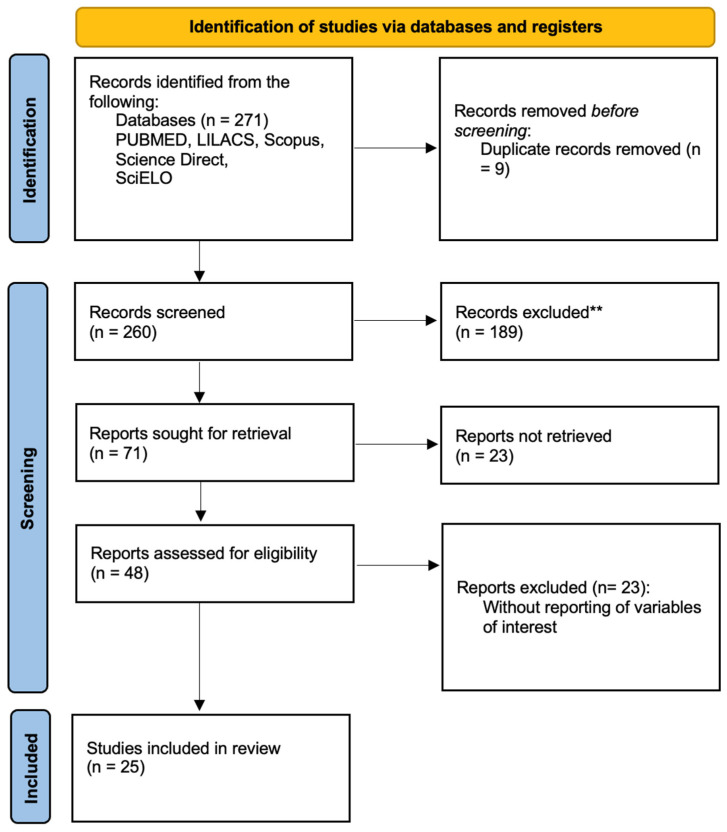
Prisma diagram of the systematic review.

**Figure 4:**
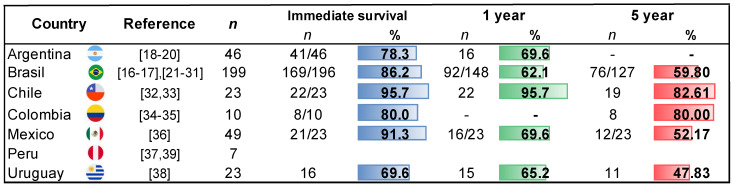
Systematic review of immediate, one-year, and five-year survival rates after heart transplantation in pediatric patients from Latin American and Caribbean countries.

The review included 25 published articles from LAC [17,18,19,20,21,22,23,24,25,26,27,28,29,30,31,32,33,34,35,36,37,38,39,40] (Figure 3), with 456 pediatric patients who underwent an HT (Figure 4). The main reason for the transplants was dilated cardiomyopathy in 58.99% (*n* = 269). The overall one-year survival rate was 88.75%; it was 66.72% at five years; and 38% were still alive after ten years (Figure 4).

## 4. Discussion

The results of this study show differences in the survival rate of pediatric patients who received an HT compared to the latest ISHLT registry era (2000–2017), with a 14.7% survival rate in the first year and a 42.6% survival rate at five years [4]. However, based on the systematic literature review from Latin American and Caribbean countries, Mexico ranks second in the frequency of HTs in childhood, representing 25% of the first-place country, Brazil.

This single-center cohort study was conducted at a national health institute in Mexico, accounting for 42% of the national database for the HT program. The median number of surgical events performed at this center is ten every five years, which, according to the systematic review by Pettit et al., classifies it as a low-volume center (fewer than ten transplants per year) [40]. Other specific situations at the hospital center include the following: (a) It is part of the Ministry of Health, which limits access to organs at the time of procurement, as the logistics of the coordination center restrict the distribution among health systems (for example, social security, state employee security, and private hospitals). (b) The location in Mexico City may increase the distance and cold ischemia time for the hospitals that procure the organs. (c) There is limited access for the hospital to offer bridge therapy. (d) The population served at our institute is the most vulnerable in terms of socioeconomic issues. These conditions stray away from the ideal reported by Killian [41] based on the results of the UNOS study (acronym) that identifies the variables associated with survival as being the donor procurement process and bridge therapy. Di Chiacchio [42] published a study that evaluated the effect of distance conditions and ischemia times on the success rate of CT during the immediate postoperative period. In our population, comparisons of the time of cold ischemia and the time of extracorporeal bypass showed statistical differences between patients who died before the first year and the rest of them.

Regarding the SDHs in the personal context, no differences were found between sex and the etiology of the disease that caused heart failure. Better survival has been reported in cardiomyopathies [43] compared to CC [4,6].

In the modified disease history of patients with cardiac malformations, the more significant manipulation of the surgical bed, cumulative damage to other organs, and a greater exposure to blood transfusions increased the risk of rejection of the donated organ [5]. In our series, children with CC who underwent transplantation had received a median of three previous cavopulmonary shunt procedures. In addition, patients with complex situations such as re-transplantation, multi-organ transplantation, or syndromic associations were not included [44,45].

The median waiting time on the transplant list was 12 months, and less than 15% were performed in the “zero” urgency context. These conditions reflect that the patients were exposed to a period of prolonged heart failure that could have deteriorated the ideal health status for transplantation, perpetuating the baseline conditions of hypoperfusion (to other organs such as the liver and kidneys) with exacerbations, leading to re-hospitalizations during HF exacerbation, and causing a deterioration in the nutritional status [46,47].

Regarding ethical considerations in access to health, authors such as Leshman [48] and Amdani [49,50] have reported that there is an unfavorable outcome in transplant patients of the Black and Latino races and in those who have a low socioeconomic status. Although racial characteristics are undifferentiated in Mexico, this study had an inclusion bias, since it was conducted in a center that serves a population without social security, that is, those who have informal employment and are at the minimum wage line.

In the analysis of risk conditions associated with death during the first year, we identified two variables related to the personal context, which were suboptimal immunosuppression due to poor attachment (27%) and the persistence of malnutrition (38.6%), which is a consequence of the absence of clinical improvement after CT. In this regard, J. Anthony et al. [51] reported that the persistence of nutritional status alterations in post-transplant patients is associated with unfavorable outcomes mediated by social conditions, the rejection of the transplanted organ, and comorbidities that may ultimately lead to death.

In pediatrics, the idea that minors’ autonomy and decision-making capacity are delegated to the primary caregivers (PC), who, together with health personnel, must make decisions based on the best and supreme interest of the minor, should be prioritized [52]. In this regard, our cohort showed variables that are related to family social circumstances; for example, 18.4% of the CPs had higher education, 84% of the families reported an income less than USD 1000 per month, and 23.7% of these incomes were due to informal employment; in addition, 58% lived at a distance greater than 51 km from the hospital. The above coincides with what was reported by Triplett et al. [41], who analyzed the direct impact that the circumstances of a CP have on the success of their child’s HT and concluded that overload, the late identification of complications, and a poor therapeutic adherence to support measures are associated with unfavorable outcomes. In our cohort, 50% of the patients developed moderate to severe rejection. This variable conditioned a 4.5 times higher risk (HR) for death after the first year. Other variables identified in the model were the parents’ education and economic income. These data coincide with other works, such as those of Olivia [53], Brown [54], and Mehta [55], who have published statistical associations in prediction models between a social environment with a poverty status and the presence of organ rejection.

It should be remembered that the objective of an HT is to reintegrate children into daily life so that they can be self-sufficient and lead a lifestyle like that of their peers. In our cohort, 65.8% of the transplanted patients returned to school, which is lower than in other series; for example, J. Anthony [51,56] reported that, in a cohort of children who received an HT, 100% returned to school and 93% returned to physical activity.

It is essential to consider that HT programs must be multidisciplinary in all areas to guarantee bio-psycho-social wellbeing, since this will provide better therapeutic adherence and a healthy lifestyle in the future. In this regard, it is essential to mention that two of the adolescent patients who were successfully transplanted attempted suicide, which reflects a failure in mental health care. Donald et al. [57] conducted a cohort study of 72 pediatric heart transplant recipients, identifying the risk factors associated with mortality during the transition to adulthood. During this period, 27.7% of the patients reported substance abuse, while 29.2% exhibited a poor adherence to immunosuppressive therapy. Additionally, psychiatric disorders were associated with a significantly increased risk of mortality (HR = 45.3). In our cohort, we identified the structural limitation of being a pediatric-exclusive center, which is the restriction of a seamless referral to appropriately equipped adult care facilities. In Mexico, structured transition clinics for adolescent and young adult transplant recipients remain unavailable, underscoring the need for integrated transplant programs that incorporate comprehensive mental health support and well-defined referral pathways to adult care. This gap in continuity of care may similarly impact other pediatric transplant centers across Latin America and the Caribbean [57].

In Mexico, most hospitals with specialized training in the care of CHD are exclusively pediatric centers. This implies that, when the patients come of age (over 18 years in Mexico), they must look for general or highly specialized hospitals with clinical cardiology clinics, which do not necessarily have health personnel trained in the transitional care of cardiac malformations [58]. Patients who are cared for by the Ministry of Health regime are more likely to have losses in follow-up during adulthood, a period during which therapeutic adherence is compromised. Rodena et al. [59] showed that, in addition to the immunosuppression monitoring of patients with CT, there is an increased risk of developing coronary disease and presenting with cardiovascular events. Another situation to consider is the effect that growth has on organ survival. Burnstein [60] published that patients with HF who were transplanted in adulthood have a higher survival rate for the donated heart compared to those who were transplanted during childhood, primarily due to the size of the organ and because there is a greater probability of a match in chronological age between the donor and the recipient. There is also the factor that the median survival rate of a transplanted heart is ten years, and that re-transplantation is a possibility in those patients who were transplanted at earlier ages; specifically, Mexico performed its first re-transplant in 2021 in a patient with ischemic heart disease [61].

Kirk [62] published a paper comparing CT survival in five periods or eras (1982, 1990, 2004, and 2009), showing an improvement in death-free periods associated with different innovations that significantly impacted the care of early complications. Our center’s experience covers the last two periods. During the five periods analyzed, the survival rate was similar, except for the years 2016–2023; the authors justified this by the restructuring of the hospital hosting the study due to the SARS-CoV-2 virus pandemic that forced specialized centers to care for all patients (of any age group) with severe COVID-19 [63].

Other institutional conditions, such as waiting times on the CT list, administrative and economic limitations to the access of bridging therapy, and the center’s experience due to the small number of transplants per year, may also account for these results. Because most deaths occur between the first and fifth year, our results weigh the influence of SDHs on the long-term survival.

Another aspect of SDHs is the responsibility of the environment and the reality of the country where the patient is located. There are marked differences in the socioeconomic conditions in developing countries, North America, and Europe, so logistic and therapeutic standards should not be universalized because they do not solve the underlying problems. The systematic review of the literature for the countries of Central America, South America, and the Caribbean (LAC) allowed us to determine that, when comparing the results of North America (USA and Canada), the number of children transplanted in 8 years was 5.9 times higher (3586 vs. 604) than the sum of the entire history of HTs in the rest of the continent; in addition, only 15% of LAC countries offer the possibility to children with HF to enter an HT program.

In 2014, the Pan-American Health Organization (PAHO) proposed that, to achieve more significant equity in access to health, PAHO member countries should increase their public expenditure on health (PHE) to at least 6% of their gross domestic product (GDP). The PHE has been widely studied and is associated with the social determinants of health. Studies have even confirmed the relationship between improvements in health and an increase in the PHE, since this implies a greater availability of resources for health care. According to a report published in 2022 by the PAHO, Mexico had a PHE of 3.3%, which is below countries such as the USA (10.68%) and Canada (9.7%). The international recommendation is that LAC receives at least 6% of its GDP [64]. This means that an LAC inhabitant has a disadvantage from birth in accessing ideal treatment conditions for catastrophic diseases such as those that lead to heart failure. The above conditions are a cohesion of factors that condition organizational inequity, in which the results for the effectiveness of health systems are explained by other components such as education, economy, and politics, which ultimately result in the same individual with a specific condition experiencing different results. On the subject of pediatric HTs, the limitation in the prompt detection of diseases that cause heart failure is the education of health personnel and society to favor the promotion of the culture of organ donation and its care intervention. Economically, health systems should generate a channel to avoid bureaucracy that limits access to all types of resources and that facilitates their rapid availability. The entire process is expensive and represents a burden for public systems (the state) or for family members (when private care is provided and expenses are out-of-pocket). One proposed alternative is the integration of funds to allow the entry of donations from third parties, associations, and own resources (from research) to avoid overloading the public sector. In addition, HT programs should adhere to outcome indicators and not process indicators that allow for continuous improvement.

In our review, Brazil had the highest number of pediatric CT reports, with 199 (56%) and a one-year survival rate of 62%. The following country was Mexico, which performed 49 transplants, corresponding to 14%. Although this figure is considerably lower than Brazil’s, Mexico achieved better survival rates of 70% for 1 year. Chile was the LAC country with the highest survival rate, with 95% one-year survival for a total of 23 pediatric patients who underwent a transplant, followed by Argentina, with 70% survival for 46 pediatric transplant patients. All these countries have GDPs of less than 6%.

Based on the above, the authors identified the following reflections.

Social inequality in Latin American countries makes it challenging to achieve universal health coverage (UHC), which is necessary to achieve the sustainable development goal on health and wellbeing. The care of highly complex diseases with long evolution times requires social programs aimed at supporting these people and their primary caregivers to avoid burnout and the consequent therapeutic detachment or abandonment. In Mexico, integrating a single health system has been a challenge, with inefficient results over the last thirty years. Specifically, the management of programs such as organ transplants could improve with articulation between the different systems, which could be more efficient by strengthening each of them to avoid lost opportunities for care.

Although preventive medicine programs do not apply to patients who have had transplants, once they successfully receive the organ, an opportunity is presented to them where a new objective of comprehensive preventive medicine could be applied, allowing for the incorporation of healthy lifestyles, including mental health care, ultimately resulting in the increased survival of the transplanted organ. Specifically to HTs, the recipients require prevention to avoid the development of cardiovascular disease and neoplasia.

Patients who have received transplants during the pediatric stage require, as a right, clinics that can enable their transition to adulthood so that, in an assisted manner, the “new adults” acquire active participatory responsibility of their self-care and the loss of continuity in standard pharmacological therapy is prevented, in order to avoid rejection and the complications associated with it. At the same time, in an assisted manner, they can be advised in the following stages of life, such as the incorporation of physical activity, work life, and reproductive health.

The retrospective nature of our data collection limited the evaluation of key variables, including the impact of antithrombotic or anticoagulant therapy on the long-term thrombotic risk. Venous thromboembolism (VTE) is a recognized complication post-heart transplantation, with a reported cumulative incidence of 42% within 60 days. Given its high recurrence, long-term anticoagulation is warranted in patients with prior VTE [65]. Additionally, a CTA-derived peri-coronary fat attenuation index has been validated for predicting allograft rejection and cardiovascular events. However, its limited availability at our center during the study period restricted its role in early rejection surveillance [66].

A possible solution to mitigate the deleterious effects of SDHs in Mexico is the incorporation into the 2020–2024 Health Sector Program, which proposed a new care model where primary health care (PHC) is resumed and integrated service networks are built to achieve a change in the health system by optimizing financial, human, and infrastructure resources [62] that can reduce social inequalities in health care and focus on the social determinants of health that represent a poor prognosis, in order to improve the survival of patients undergoing an HT.

The strength of this study lies in the fact that its population represents 42% of the HTs performed in Mexico’s pediatric stage. It is the only published report that approximates a national representation of the results and the only one to explore SDHs.

We recognize the following methodological weaknesses of this study:

(a) The sample size was minimal, and many variables were measured, which limited the performance of the statistical analysis in adjusting for confounding variables.
(b) One of the study’s limitations was the data collection process, which spanned over 20 years and relied on records. This method carries inherent risks of bias due to the misclassification and memory of risk variables, particularly SDHs.
(c) It is possible that variables currently associated with immediate and mediate mortality, such as biomarkers, viral infections, and variables that measure the immune response, were not measured, because they were not measured consistently during the first decade of the institution’s transplant program.

Regarding the systematic literature review, the variables measured in the included articles were inconsistently reported and heterogeneous in their definition, making a meta-analysis impossible.

One way to improve the external validity of the results is to expand this research to a multicenter collaboration.

## 5. Conclusions

This cohort of pediatric patients who received an HT enabled the identification of risk variables of personal and community SDHs for mortality in the first and fifth years. Chronic rejection occurred in 50%, and the absence of transitional care to adulthood was the variable with the highest risk. The systematic review identified Mexico as the country with the second-highest frequency of CT, and our cohort accounted for 42% of the total number of transplants in the country. Numerically, LAC has a lower frequency of transplants and survival in the first year compared to other developed countries, possibly due to a gap associated with organizational justice.

## Figures and Tables

**Table 1:** General characteristics of the cohort of pediatric heart transplant patients.

Gender	*n*	%
Men	22	58%
Women	16	42%
Pediatric Stage		
Toddler	6	16%
Preschool	9	24%
Grade-schooler	12	32%
Adolescent	11	28%
Etiology		
Congenital heart disease	18	47%
Cardiomyopathies	20	53%
Malnutrition before transplant	34	89.5%
Waiting time for heart transplant (months)	12	6–12
High-urgency waiting status	6	15.8%
Blood Group		
O	13	34%
A	20	52%
B	5	13%
AB	1	3%
Heart Rate After Transplant		
Sinusal	37	97%
Atrioventricular block	1	3%
Cause of Death in the First Year (n = 9)		
Heart failure	5	14%
Septic shock	1	3%
Acute rejection	4	11%

**Table 2:** Surgical variables of the cohort of pediatric heart transplant patients.

Variables	Total			Non-Survivors			Survivors		
	Median	p25	p75	Median	p25	p75	Median	p25	p75
Age at transplant (years)	88	55	122	85	23	131	72	52	123
Cold ischemia (min) **	260	167	308	275	235	315	169	165	250
Aortic clamping (min)	120	80	170	120	90	170	130	80	176
Extracorporeal bypass (min)	138	115	150	123	115	150	148	125	160
Hospital stay (days)	19	11	30	16	11	23	23	18	56
Survival after heart transplant rejection (days)	451	44	1979	1979	364	2260	198	33	451
Survival after transplant (years) **	7	4	12	2	1	5	8	2	16

Mann–Whitney test, ** *p*-value ≤0.001.

**Table 3:** Social determinants of health in pediatric heart transplant patients, by period.

	Total		2001–2005		2006–2010		2011–2015		2016–2022	
	*n* = 38		*n* = 12		*n* = 6		*n* = 13		*n* = 7	
	*n*	%	*n*	%	*n*	%	*n*	%	*n*	%
Personal Social Factors										
Sex										
Men	22	57.9%	7	58.30%	4	66.70%	6	46.20%	5	66.60%
Women	16	42.1%	5	41.70%	2	33.30%	7	53.80%	2	33.40%
Age at heart transplant (months)			74	42–99	108	83–123	84	52–131	96	90–120
Malnutrition before transplant	34	89.5%	10	83%	6	100%	11	85%	7	100%
Waiting time to transplant (months)	12		13	7–15	12	9–18	10	1–15	15	2–17
High-urgency waiting status	6	15.8%	2	17%	1	17%	2	15%	1	14%
Diagnosis										
Congenital heart disease	18	47.4%	6	50%	2	33%	6	46%	4	57%
Myocardiopathies	20	52.6%	6	50%	4	67%	7	54%	3	43%
Discharge treatment										
Optimal immunosuppression	24	63.2%	8	67%	4	67%	8	62%	4	57%
Optimal heart failure medication	33	86.8%	10	83%	6	100%	10	77%	7	100%
Persistence of malnutrition at one year	14	36.8%	5	42%	3	50%	4	31%	2	33%
Depression	8	21.1%	1	8%	2	33%	5	39%	0	0%
Back to school	25	65.8%	9	75%	4	67%	10	77%	2	33%
Rejection	19	50.0%	8	62%	3	50%	7	54%	1	17%
Family Social Factors										
Distance between home and transplantation center										
<50 km	16	42.1%	6	50%	3	50%	5	38%	2	29%
51–100 km	12	31.6%	3	25%	2	33%	4	31%	3	43%
101–300 km	6	15.8%	2	17%	1	17%	2	15%	1	14%
>300 km	2	5.3%	0	0%	0	0%	1	8%	1	14%
Education of primary caregivers										
Elementary school	3	7.9%	1	8%	1	17%	1	8%	0	0%
Middle school	6	15.8%	2	17%	1	17%	2	15%	1	14%
High school	15	39.5%	5	42%	2	33%	5	38%	3	43%
Technical college	7	18.4%	1	8%	2	33%	3	23%	1	14%
College university	7	18.4%	3	25%	0	0%	2	15%	2	29%
Socioeconomic level (INEGI)										
A/B level	0	0.0%	0	0%	0	0%	0	0%	0	0%
C level	6	15.8%	2	17%	1	17%	2	15%	1	14%
D level	26	68.4%	7	58%	4	67%	10	77%	5	71%
E level	6	15.8%	3	25%	1	17%	1	8%	1	14%
Informal employment	9	23.7%	3	25%	2	33%	2	15%	2	29%
Community Factors and Access to Health Services										
Transitional care to adulthood	13	34.2%	6	50%	4	67%	3	23%		
Social security	10	26.3%	5	42%	1	17%	4	31%		
Survival										
1 year	29	76.3%	9	75%	4	66%	11	85%	5	71%
5 years	18	47.4%	6	50%	2	33%	8	62%	2	29%
10 years	6	15.8%	1	8%	1	17%	4	31%	0	0%

## Data Availability

The datasets for this study are available by contacting the corresponding author.

## Supplementary Materials

The following supporting information can be downloaded at: https://www.mdpi.com/article/10.3390/jcm14051506/s1. Figure S1: Search strategies.

## References

[1] Greenberg J.W., Guzman-Gomez A., Hogue S., Morales D.L.S. (2022). Pediatric heart transplantation: The past, the present, and the future. Semin. Pediatr. Surg..

[2] Atik F.A., Couto C.F., Tirado F.P., Moraes C.S., Chaves R.B., Viera N.W., Reis J.G. (2014). Addition of long-distance heart procurement promotes changes in heart transplant waiting list status. Rev. Bras. Cir. Cardiovasc..

[3] Jaiswal A., Baran D.A. (2024). Moving Beyond the Ice Age in Heart Transplant Procurement. Transplantation.

[4] International Thoracic Organ Transplant (TTX) Registry|ISHLT. https://www.ishlt.org/registries/international-thoracic-organ-transplant-(ttx)-registry.

[5] Shah M.J., Silka M.J., Silva J.N.A., Balaji S., Beach C.M., Benjamin M.N., Berul C.I., Cannon B., Cecchin F., Cohen M.I. (2021). 2021 PACES Expert Consensus Statement on the Indications and Management of Cardiovascular Implantable Electronic Devices in Pediatric Patients: Developed in collaboration with and endorsed by the Heart Rhythm Society (HRS), the American College of Cardiology (ACC), the American Heart Association (AHA), and the Association for European Paediatric and Congenital Cardiology (AEPC) Endorsed by the Asia Pacific Heart Rhythm Society (APHRS), the Indian Heart Rhythm Society (IHRS), and the Latin Americ. JACC Clin. Electrophysiol..

[6] Hahn E.A., Allen L.A., Lee C.S., Denfeld Q.E., Stehlik J., Cella D., Lindenfeld J., Teuteberg J.J., Mcilvennan C.K., Kiernan M.S. (2023). PROMIS: Physical, Mental and Social Health Outcomes Improve From Before to Early After LVAD Implant: Findings from the Mechanical Circulatory Support: Measures of Adjustment and Quality of Life (MCS A-QOL) Study. J. Card. Fail..

[7] Lin C., Cousins S.J., Zhu Y., Cingan S.E., Monney L.J., Kan E., Wu F., Hser Y.I. (2024). A scoping review of social determinants of health’s impact on substance use disorders over the life course. J. Subst. Use Addict. Treat..

[8] Cáceres Rivera D.I., Paredes Suárez S.M., Cardenas Herrera M.S., Ruiz Sandoval J.P., Rincón Romero M.K., López-Romero L.A. (2024). Parameters for delivering ethnically and gender-sensitive primary care in cardiovascular health through telehealth. Systematic review. Public Health.

[9] Argüero-Sánchez R., Sánchez-Ramírez O., Olivares-Durán E.M. (2020). D transplantation eceased donation and organ in Mexico. Analysis of 12 years and seven strategic proposals. Cirugía Cir..

[10] Centro Nacional de Trasplantes|Gobierno|gob.mx. https://www.gob.mx/cenatra/.

[11] Operational Framework for Monitoring Social Determinants of Health Equity. https://www.who.int/publications/i/item/9789240088320.

[12] Ludhwani D., Abraham J., Sharma S., Kanmanthareddy A. (2024). Heart Transplantation Rejection.

[13] Standards. https://www.who.int/tools/child-growth-standards/standards.

[14] Encuesta Nacional de Ingresos y Gastos de los Hogares 2020. https://www.inegi.org.mx/programas/enigh/nc/2020/.

[15] Page M.J., McKenzie J.E., Bossuyt P.M., Boutron I., Hoffmann T.C., Mulrow M.D., Shamseer L., Tetzlaff J.M., Akl E.A., Brenan S.E. (2021). The PRISMA 2020 statement: An updated guideline for reporting systematic reviews. PLoS Med..

[16] von Elm E., Altman D.G., Egger M., Pocock S.J., Gøtzsche P.C., Vandenbroucke J.P. (2008). The Strengthening the Reporting of Observational Studies in Epidemiology (STROBE) statement: Guidelines for reporting observational studies. J. Clin. Epidemiol..

[17] Sabbaga Amato M., Amato Neto V., Uip D.E. (1997). Avaliação da Qualidade de Vida de Pacientes com Doença de Chagas Submetidos a Transplante de Coração. Rev. Soc. Bras. Med. Trop..

[18] Natalia P., Lanura B., Matsubara L., Neto V.A., Okumura Edimar M., Bocchi A. (1995). Caracterização de Cepas do Trypanosoma Cruzi Isoladas de Doentes nos Quais foi Realizado Transplante de Coração. Rev. Soc. Bras. Med. Trop..

[19] Peradejordi M., Favaloro Mtsac R.R., Bertolotti A., Abud J., Gómez C., Absi D., Favaloro L.M., Martinez L. (2011). Moscoloni, S. Trasplante Cardíaco Ortotópico: Resultados Del Hospital Universitario Fundación Favaloro Orthotopic Heart Transplantation: Results from the Hospital Universitario Fundación Favaloro. Rev. Argent. Cardiología.

[20] Vogelfang H., Naiman G., Villa A., Moreno G., Charroqui A., Quiroga L., Burlli G., Magliola R., De la Riba M.C. (2009). Ocho Años de Experiencia En Trasplante Cardiaco Infantil En El Hospital Juan, P. Garrahan. https://pesquisa.bvsalud.org/portal/resource/pt/lil-538112?src=similardocs.

[21] Juffe A. (2011). Trasplante Cardíaco lecciones aprendidas. Rev. Argent. Cardiol..

[22] dos Santos A.A., da Silva J.P., da Fonseca L., Baumgratz J.F. (2012). Retransplante cardíaco em criança sem o uso de hemoderivados. Braz. J. Cardiovasc. Surg..

[23] de Sylos C., Azeka E., Kajita L., Bevenutti L., Casaro Strunz C., Castello Branco K., Almeida Riso A., Tanamati C., Jatene M., Barbero Marcial M. (2009). Evaluación Del Péptido Natriurético Tipo B En El Diagnóstico de Rechazo Tras Transplante Cardiaco Pediátrico. Arq. Bras. Cardiol..

[24] Croti U.A., Marcolino Braile D., Moscardini A.C., Leiroz A.C., Botelho F., Kozak M. (2010). Heart Transplantation in Child with Non-Compacted. Braz. J. Cardiovasc. Surg..

[25] Biscegli Jatene M., Augusto Miana L., John Pessoa A., Riso A., Azeka E., Tanamati C., Solange G., Augusto Lopez A., Barbero Marcial M., Groppo Stolf N.A. (2007). Pediatric Heart Transplantation in Refractory Cardiogenic Shock: A Critical Analysis of Feasibility, Applicability and Results. Arq. Bras. Cardiol..

[26] Carlos R.V., Torres M.L.A., de Boer H.D. (2018). Reversal of neuromuscular block with sugammadex in five heart transplant pediatric recipients. Braz. J. Anesthesiol. (Engl. Ed.).

[27] Riva N., Cáceres Guido P., Licciardone N., Imventarza O., Monteverde M.L., Staciuk R., Charroqui A., Schaiquevich P. (2017). Therapeutic monitoring of pediatric transplant patients with conversion to generic tacrolimus. Farm. Hosp..

[28] Miana L.A., Azeka E., Canêo L.F., Turquetto A.L., Tanamati C., Penha J.G., Cauduro A., Jatene M.B. (2014). Pediatric and congenital heart transplant: Twenty-year experience in a tertiary Brazilian hospital. Braz. J. Cardiovasc. Surg..

[29] Caneo L.F., Miana L.A., Jatene M.B. (2016). Circulatory support as a bridge to pediatric heart transplantation. Arq. Bras. Cardiol..

[30] Pego Fernandes M.P., Azeka E., Odoni V., Machado Junqueira J.J., Paiva Bento G., Aiello V., Barbero Marcial M. (2006). Case Report Post-Transplantation Lymphoproliferative Disorder in a Pediatric Patient. Arq. Bras. Cardiol..

[31] Canêo L.F., Miana L.A., Tanamati C., Gomes Penha J., Satsuki Shimoda M., Azeka E., Miura N., Barbosa Gomes Galas F.R., Alves Guimaraes V., Biscegli Jatene M. (2015). Use of short-term circulatory support as a bridge in pediatric heart transplantation. Arq. Bras. Cardiol..

[32] Becker P.R., Besa S., Riveros S., González R., Navia A., Dellepiane P., Springmuller D., Urcelay G. (2017). Resultados de un programa nacional de trasplante cardiaco pediátrico: Fortalezas y debilidades. Rev. Chil. Pediatr..

[33] Trincado C., Molina V., Urcelay G., Dellepiane P. (2018). Echocardiographic evaluation after pediatric heart transplant in Chile: Initial application of a functional protocol with global longitudinal strain. Rev. Chil. Pediatr..

[34] Zuluaga Aristizábal I.C., Restrepo Bernal D.P., Zuluaga Restrepo M., Zapata Sánchez M.M., Palomino Rodríguez A.A. (2021). Encefalopatía posterior reversible en niño trasplantado de corazón. CES Med..

[35] Castillo V.R., Jaramillo G.A., Hernández A., Andrade O.H., Salazar L., Luna H.J., Durán A.E. (2006). Transplante cardiaco en niños: Reporte del primer caso atendido en la Fundación Cardiovascular de Colombia. Rev. Colomb. Cardiología.

[36] Cerdán A.B., Ruiz González S., Santos Monter M.J. (2013). Diez años de trasplante cardiaco en Pediatría: Experiencia en el Hospital Infantil de México Federico Gómez. Boletín Médico Hosp. Infant. México.

[37] Skrabonja-Crespo A., Chavarri-Velarde F., Pinto-Salinas M., Tauma-Arrué A. (2020). Percutaneous endovascular management of ascending aortic pseudoaneurysm after heart transplantation in a pediatric patient. Pediatr. Transplant..

[38] Ceruti B., Chiesa P., Tambasco J., Anzibar R., Gutiérrez C., Barboza S., Manfredi A., Leone R. (2012). Trasplante cardíaco. Experiencia de 15 años del ICI Trasplante cardíaco Experiencia de 15 años del Instituto de Cardiología Infantil. Rev. Urug. Cardiología.

[39] Aguilar Carranza C., Alarco W., Soplopuco F., Moron j., Lescano M., Morales J., Galvez D. (2018). Pathology of heart transplantation in peru: Experience with 61 cases in a national reference center. Rev. Peru. Med. Exp. Salud Publica.

[40] Pettit S.J., Jhund P.S., Hawkins N.M., Gardner R.S., Haj Yahia S., McMurray J.J.V., Petrie M.C. (2012). How small is too small? A systematic review of center volume and outcome after cardiac transplantation. Circ. Cardiovasc. Qual. Outcomes.

[41] Killian M.O., Little C.W., Howry S.K., Watkivs M., Triplett K.N., Desai D.M. (2024). Demographic Factors, Medication Adherence, and Post-transplant Health Outcomes: A Longitudinal Multilevel Modeling Approach. J. Clin. Psychol. Med. Settings.

[42] DiChiacchio L., Goodwin M.L., Kagawa H., Griffiths E., Nickel I.C., Stehlik J., Selzman C.H. (2023). Heart Transplant and Donors After Circulatory Death: A Clinical-Preclinical Systematic Review. J. Surg. Res..

[43] Bearl D.W. (2019). Ethical issues in access, listing and regulation of pediatric heart transplantation. Transl. Pediatr..

[44] Lotan D., DeFilippis E.M., Oren D., Vinogradsky A., Rubinstein G., Mathur A., Takeda K., Hua M., Gaglio P.J., Szabolcs M.J. (2023). Combined heart and liver transplantation in a patient supported by left ventricular assist device (LVAD) with propionic acidemia. Nutr. Metab. Cardiovasc. Dis..

[45] Auerbach S.R., Arshad A., Azeka E., Cantor R.S., Kirklin J.K., Koehl D., Menteer J., Peng D.M., Ravekes W., Shaw F.R. (2024). Impact of prolonged ischemic time on pediatric heart transplantation outcomes: Improved outcomes in the most recent era. J. Heart Lung Transplant..

[46] McDonagh T.A., Metra M., Adamo M., Gardner R.S., Baumbach A., Böhm M., Burri H., Butler J., Celutkiené J., Chioncel O. (2023). 2023 Focused Update of the 2021 ESC Guidelines for the diagnosis and treatment of acute and chronic heart failure. Eur. Heart J..

[47] McDonagh T.A., Metra M., Adamo M., Gardner R.S., Baumbach A., Böhm M., Burri H., Butler J., Celutkiene J., Chioncel O. (2021). 2021 ESC Guidelines for the diagnosis and treatment of acute and chronic heart failure. Eur. Heart J..

[48] Lehman L.L., Mostofsky E., Salia S., Gupta S., Barrera F.J., Liou L., Mittleman M.A. (2022). Racial and Ethnic Disparities in Incidence and Prognosis of Perioperative Stroke Among Pediatric Cardiac Transplant Recipients. J. Am. Heart Assoc. Cardiovasc. Cerebrovasc. Dis..

[49] Amdani S., Gossett J.G., Chepp V., Urschel S., Asante-Korang A., Dalton J.E. (2024). Review on clinician bias and its impact on racial and socioeconomic disparities in pediatric heart transplantation. Pediatr. Transplant..

[50] Amdani S., Conway J., Kleinmahon J., Auerbach S., Hsu D., Cousino M.S., Kaufman B., Alejos J., Hopper Cruz J., Y Lee H. (2023). Race and Socioeconomic Bias in Pediatric Cardiac Transplantation. JACC Heart Fail..

[51] Anthony S.J., Pollock BarZiv S., Ng V.L. (2010). Quality of life after pediatric solid organ transplantation. Pediatr. Clin. N. Am..

[52] Willems D.L. (2001). Balancing rationalities: Gatekeeping in health care. J. Med. Ethics.

[53] Oliva M., Singh T.P., Gauvreau K., Vanderpluym C.J., Bastardi H.J., Almond C.S. (2013). Impact of medication non-adherence on survival after pediatric heart transplantation in the U.S.A. J. Heart Lung Transplant..

[54] Brown K.L., Ramaiah R., Fenton M., Wood T.M., Scott K., Carter K., Wray J., Burch M. (2009). Adverse family social circumstances and outcome in pediatric cardiac transplant recipients at a UK center. J. Heart Lung Transplant..

[55] Mehta P., Steinberg E.A., Kelly S.L., Buchanan C., Rawlinson A.R. (2017). Medication adherence among adolescent solid-organ transplant recipients: A survey of healthcare providers. Pediatr. Transplant..

[56] Anthony S.J., Annunziato R.A., Fairey E., Kelly V.L., So S., Wray J. (2014). Waiting for transplant: Physical, psychosocial, and nutritional status considerations for pediatric candidates and implications for care. Pediatr. Transplant..

[57] Donald E.M., Oren D., DeFilippis E.M., Rubinstein G., Moeller C.M., Y Lee H., Maldonado A., Portera M.V., Fuselier B., Jackson R. (2024). Long-term outcomes for pediatric heart transplant recipients transitioning to adult care teams. Clin. Transplant..

[58] Calderon-Colmenero J. (2019). Regionalization of congenital heart disease care: A pending goal. Arch. Cardiol. Mex..

[59] Rodenas-Alesina E., Aleksova N., Stubbs M., Fouroutan F., Kozuszko S., Posada J.D., McDonal M., Moayedi Y., Ross H., Dipchand A. (2024). Cardiac allograft vasculopathy and survival in pediatric heart transplant recipients transitioned to adult care. J. Heart Lung Transplant..

[60] Burstein D.S., Rossano J.W., Lindenfeld J., Schlendord K., Do N., Godown J., O’Connor M., Maeda K., Edelson J.B., Lin K.Y. (2022). Association of Donors with US Public Health Service Risk Criteria and Outcomes after Adult vs. Pediatric Cardiac Transplant. JAMA Cardiol..

[61] Careaga-Reyna G., Zetina-Tun H.J. (2018). Retrasplante cardiaco electivo. Primer caso en México. Gac. Med. Mex..

[62] Kirk R., Butts R.J., Dipchand A.I. (2019). The first successful pediatric heart transplant and results from the earliest era. Pediatr. Transplant..

[63] Ordoñez-González I., Basurto M.A. (2023). Primary health care during the COVID-19 pandemic in Mexico. Rev. Med. Inst. Mex. Seguro Soc..

[64] Pedraza C.C., Matus-López M., Báscolo E. (2018). Fiscal space for Health in the Americas: Is economic growth sufficient?. Rev. Panam. Salud Publica/Pan Am. J. Public Health.

[65] Karakasis P., Giannakoulas G., Theofilis P., Patoulias D., Fragakis N. (2024). Direct oral anticoagulants or vitamin K antagonists in adult patients with congenital heart disease?. Eur. J. Intern. Med..

[66] Sansonetti A., Belmonte M., Masetti M., Bergamaschi L., Paolisso P., Borgese L., Angeli F., Armillotta M., Dierckx R., Verstreken S. (2025). CTA-Derived Pericoronary Fat Attenuation Index Predicts Allograft Rejection and Cardiovascular Events in Heart Transplant Recipients. JACC Cardiovasc. Imaging.

