# Supplementary material for: The Influence of Social Determinants of Health on the Survival of Heart Transplants in the Pediatric Age: An Analysis of a Mexican Cohort and Its Comparison with Latin America and the Caribbean
Source: J Clin Med. 2025 Feb 24;14(5):1506. doi: 10.3390/jcm14051506 (PMC11900437; doi:10.3390/jcm14051506)
Supplement: Supplementary file 1 [file jcm-14-01506-s001.zip › jcm-3447047-supplementary.pdf]

Figure S1. title: Search strategies.

Supplementary material 1: Search strategies
"heart transplantation"[MeSH Terms] OR ("heart"[All Fields] AND "transplantation"[All Fields]) OR "heart transplantation"[All Fields] OR ("cardiac"[All Fields] AND "transplantation"[All Fields]) OR "cardiac transplantation"[All Fields] OR ("heart"[All Fields] AND "grafting"[All Fields]) OR "heart grafting"[All Fields] OR Orthotopic heart transplantation [All Fields])
AND
"latin america"[MeSH Terms] OR ("latin"[All Fields] AND "america"[All Fields]) OR "latin america"[All Fields] OR OR "Central America" [MeSH Terms] OR "South America" [MeSH Terms] OR "Caribbean Region"" [MeSH Terms] "Argentina" [MeSH Terms] OR "Bolivia" [MeSH Terms] OR "Brazil" [MeSH Terms] OR "Chile" [MeSH Terms] OR "Colombia" [MeSH Terms] OR "Ecuador" [MeSH Terms] OR "French Guiana" [MeSH Terms] OR "Guyana" [MeSH Terms] OR "Paraguay" [MeSH Terms] OR "Peru" [MeSH Terms] OR "Suriname" [MeSH Terms] OR "Uruguay" [MeSH Terms] OR "Venezuela" [MeSH Terms] "Aruba" [MeSH Terms] OR "Caribbean Netherlands" [MeSH Terms] OR "Curacao" [MeSH Terms] OR "Sint Maarten " [MeSH Terms] OR "West Indies" [MeSH Terms] OR "Antigua and Barbuda" [MeSH Terms] OR "Bahamas" [MeSH Terms] OR "Barbados " [MeSH Terms] OR "British Virgin Islands " [MeSH Terms] OR "Cuba" [MeSH Terms] OR "Dominica" [MeSH Terms] OR "Dominican Republic " [MeSH Terms] OR "Grenada" [MeSH Terms] OR "Guadeloupe" [MeSH Terms] OR "Haiti" [MeSH Terms] OR "Jamaica" [MeSH Terms] OR "Martinique" [MeSH Terms] OR "Puerto Rico" [MeSH Terms] OR "Saint Kitts and Nevis" [MeSH Terms] OR "Saint Lucia" [MeSH Terms] OR "Saint Vincent and the Grenadines" [MeSH Terms] OR "Trinidad and Tobago" [MeSH Terms] OR "United States Virgin Islands" [MeSH Terms] OR "Mexico" [MeSH Terms] ((Latin america OR Antigua y Barbuda OR Argentina OR Bahamas OR Barbados OR Belice OR Bolivia OR Brasil OR Colombia OR Costa Rica OR Cuba, Chile OR Dominica OR Ecuador OR El Salvador OR Granada OR Guatemala, Guyana OR Haití OR Honduras OR Jamaica OR México OR Nicaragua OR Panamá OR Paraguay OR Perú OR República Dominicana OR San Cristóbal y Nieves OR San Vicente y las Granadinas OR Santa Lucía OR Surinam OR Trinidad y Tobago OR Uruguay OR Venezuela [All fields]) OR
AND
"child"[MeSH Terms] OR "child"[All Fields] OR "children"[All Fields] OR "child's"[All Fields] OR "children's"[All Fields] OR "childrens"[All Fields] OR "childs"[All Fields] "paediatrics"[All Fields] OR "pediatrics"[MeSH Terms] OR "pediatrics"[All Fields] OR "paediatric"[All Fields] OR "pediatric"[All Fields]
Scopus
ALL("CARDIAC TRANSPLANTATION") OR ALL ("HEART TRANSPLANTATION")
AND
(KEYWORD , "Pediatrics") OR (KEYWORD , "Child") OR (KEYWORD , "Adolescent") OR (KEYWORD , "Infant") OR (KEYWORD , "Child, Preschool") OR (KEYWORD , "Preschool Child") OR (KEYWORD , "Infant, Newborn") OR (KEYWORD , "Newborn") OR (KEYWORD , "School Child") OR (KEYWORD, "Pediatric") OR (KEYWORD , "Children")
AND
LIMIT-TO (AFFILCOUNTRY , "Brazil") OR LIMIT-TO (AFFILCOUNTRY , "Mexico") OR LIMIT-TO (AFFILCOUNTRY , "Argentina") OR LIMIT-TO (AFFILCOUNTRY , "Portugal") OR LIMIT-TO (AFFILCOUNTRY , "Chile") OR LIMIT-TO (AFFILCOUNTRY , "Colombia") OR LIMIT-TO (AFFILCOUNTRY , "Peru") OR LIMIT-TO (AFFILCOUNTRY , "Uruguay") OR LIMIT-TO (AFFILCOUNTRY , "Panama") OR LIMIT-TO (AFFILCOUNTRY , "Venezuela") OR LIMIT-TO (AFFILCOUNTRY , "Cuba") OR LIMIT-TO (AFFILCOUNTRY , "Costa Rica") OR LIMIT-TO (AFFILCOUNTRY , "Ecuador") OR LIMIT-TO (AFFILCOUNTRY , "Puerto Rico") OR LIMIT-TO (AFFILCOUNTRY , "Haiti") OR LIMIT-TO (AFFILCOUNTRY , "Nicaragua") OR LIMIT-TO (AFFILCOUNTRY , "Paraguay") OR LIMIT-TO (AFFILCOUNTRY , "Dominican Republic") OR LIMIT-TO (AFFILCOUNTRY , "Guatemala") OR LIMIT-TO (AFFILCOUNTRY , "Bolivia"))
